# Ectopic Recurrence of a Craniopharyngioma

**DOI:** 10.7759/cureus.35988

**Published:** 2023-03-10

**Authors:** Vincent F Carfagno, Justin Rouintan, Imtiaz Ahmed

**Affiliations:** 1 Diagnostic Radiology, Midwestern University Arizona College of Osteopathic Medicine, Fountain Hills, USA; 2 Osteopathic Medicine, Scottsdale Sports Medicine Institute, Scottsdale, USA; 3 Radiology, Tempe St. Luke's Hospital, Tempe, USA

**Keywords:** corpus callosum lesion, neuroradiology, craniopharyngioma, recurrent craniopharyngioma, ectopic craniopharyngioma

## Abstract

Craniopharyngiomas are rare, solid or mixed solid, cystic tumors that generally occur in the sellar and suprasellar regions of the central nervous system along the pituitary stalk near the optic chiasm. Although benign, patients clinically present with symptoms from mass effect or the resultant increase in intracranial pressures. The recurrence rate of craniopharyngiomas is high. When present, recurrence is typically at the site of the primary tumor bed. Here, we present a case of a 27-year-old male who was found to have a suprasellar mass on enhanced CT imaging. The mass was resected and a ventriculoperitoneal shunt was placed. The lesion was identified as a craniopharyngioma, and the patient completed treatment and was discharged appropriately. Ten years later, the patient presented with headaches and on enhanced CT imaging was found to have a radiopaque mass originating from the right lateral ventricle and extending into the splenius of the corpus callosum. This new lesion was identified as a craniopharyngioma. Ectopic recurrence of a craniopharyngioma is a rare phenomenon and is thought to be the result of direct seeding along the site of surgical resection or via cerebrospinal fluid circulation.

## Introduction

Craniopharyngiomas are rare, typically benign tumors thought to originate from remnants of the craniopharyngeal duct [1]. These tumors, which occur in the sellar and suprasellar regions, display a bimodal incidence of distribution and most commonly occur between five and 15 and 45 and 60 years of age [2]. The clinical presentation includes headache, visual disturbances, endocrine abnormalities, and other various symptoms, including but not limited to nausea, vomiting, lethargy, and depression [3]. Treatment of craniopharyngiomas involves surgical resection with or without radiation treatment. There is currently some debate between gross-total resection alone versus limited resection along with irradiation therapy. However, like most interventions, the surgical approach and goals of treatment should be individualized for each patient. In the case of craniopharyngiomas, consideration should be toward factors such as age, endocrine status, and tumor morphology. Despite their benign character, these tumors do show high rates of recurrence [4]. When present, tumor recurrence is frequently seen at the origin of the primary tumor bed [5]. Ectopic recurrence may occur after resection.

## Case presentation

**Figure 1:**
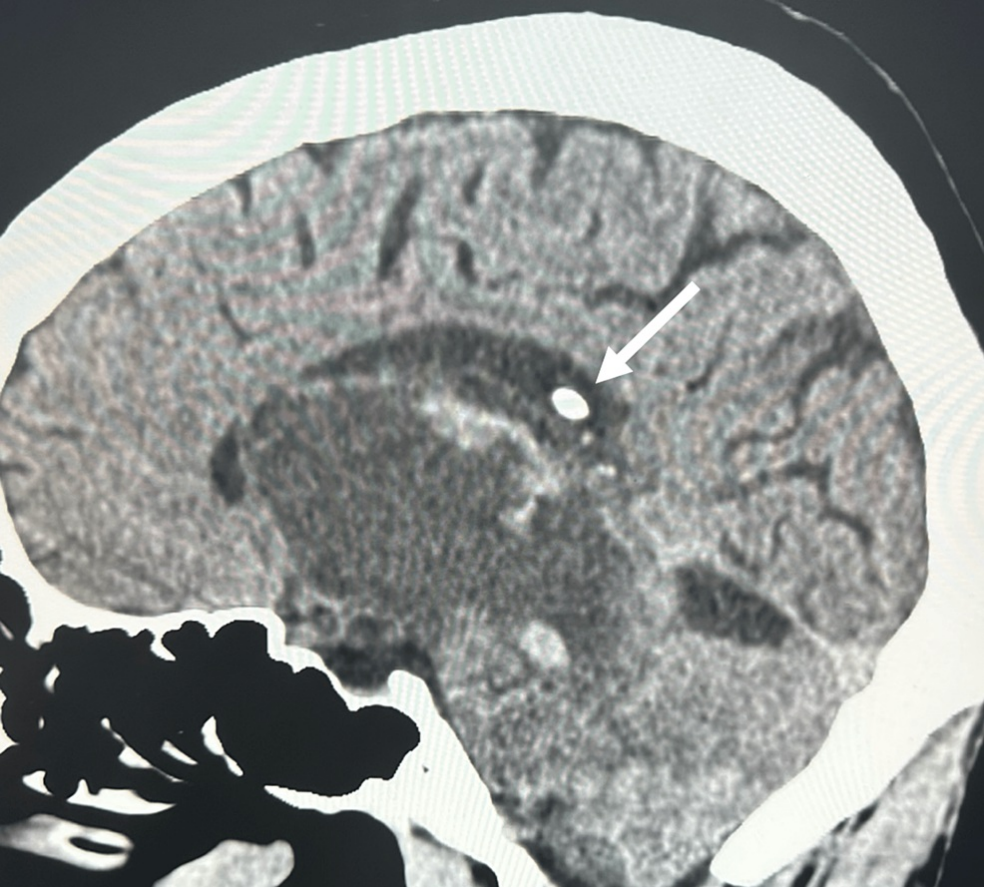
Enhanced CT, sagittal view Enhanced CT displaying a radiopaque mass, subsequently determined to be a craniopharyngioma projecting into the splenius of the corpus callosum from the body of the right lateral ventricle

**Figure 2:**
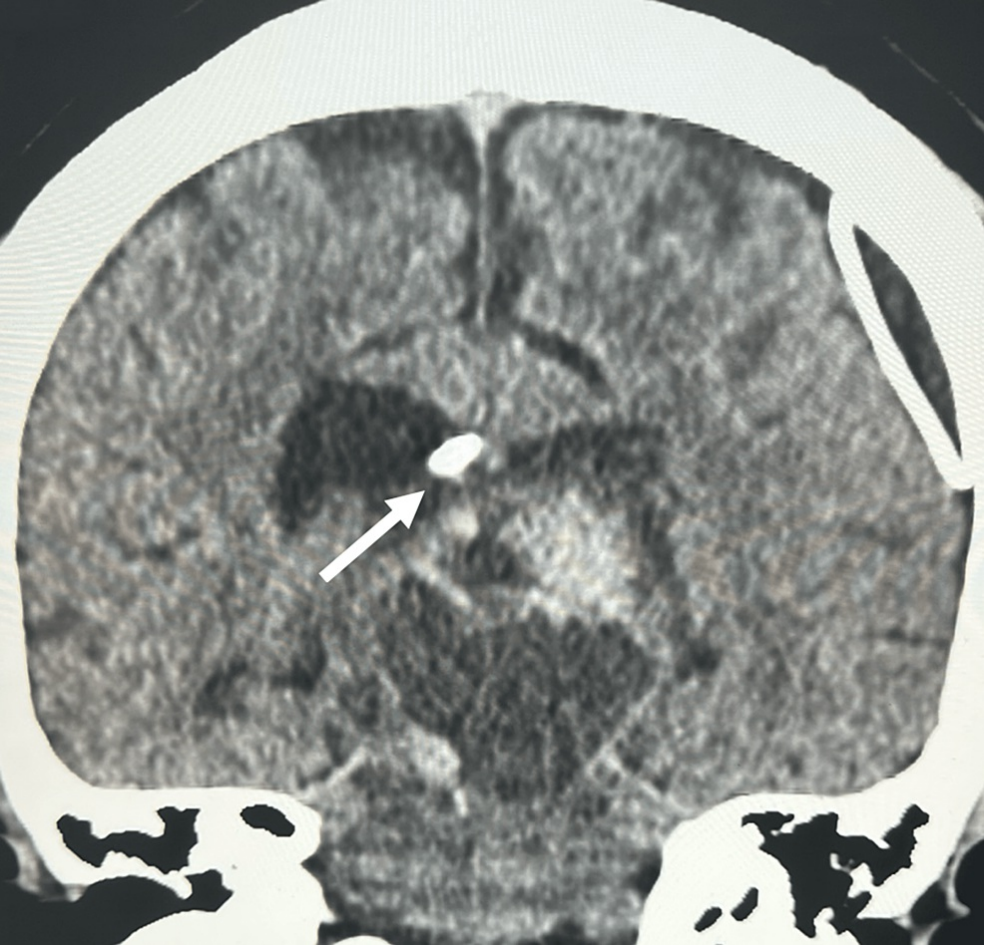
Enhanced CT, coronal view Enhanced CT displaying an additional view of the radiopaque mass extending into the splenius of the corpus callosum from the body of the right lateral ventricle

We present a 27-year-old male with a past medical history of a suprasellar craniopharyngioma treated with total resection and ventriculoperitoneal shunt placement 10 years prior who presented with a one-month history of a persistent headache. There were no noted aggravating or alleviating factors to the patient’s symptoms. He denied any other past medical history. Contrast-enhanced computed tomography (CECT) was performed on evaluation, demonstrating a radiopaque mass projecting from the body of the right lateral ventricle into the splenius of the corpus callosum (Figures 1, 2). A proximal shunt was visualized with placement within the left lateral ventricle. The patient underwent gross total resection of the lesion by a transcortical approach. There were no intraoperative complications. The patient had an uneventful postoperative course and was discharged after three days. Analysis of the lesion identified the mass to be histologically consistent with a craniopharyngioma.

## Discussion

Craniopharyngiomas are rare, with an incidence of 0.16 per 100,000 persons [6]. These tumors have a bimodal distribution of incidence, occurring between five and 15 and 45 and 60 years of age [2]. Although typically benign, the treatment of craniopharyngiomas involves total resection of the tumor to alleviate any associated symptoms that may present due to a mass effect [5]. Adjuvant radiosurgery and radiotherapy may also aid with the elimination of tumor burden [4].

Headache and visual deficits are the two most common symptoms reported in craniopharyngioma patients, which may be a result of mass effect and/or the resultant increase in intracranial pressure seen in patients [7]. Craniopharyngiomas, which frequently occur in the sellar and suprasellar regions, may also present with endocrine abnormalities given the proximity of these regions to the hypophyseal-portal system.

The recurrence rate of craniopharyngiomas is high. A previous case series identified a recurrence rate of 39.1%, with recurrence more commonly seen at the origin of the primary tumor bed [4,5]. Ectopic recurrence after tumor resection is a rare phenomenon that may occur by either direct seeding along the surgical resection pathway or via flow within the cerebrospinal fluid circulation [5]. This case described is unique in that ectopic occurrence was observed abutting the right lateral ventricle, with tumor extension into the splenius of the corpus callosum.

As discussed, craniopharyngiomas are typically benign. Treatment, however, typically involves complete surgical resection given their primary occurrence around the sellar and suprasellar areas, highly neurovascular regions known for their various vital functions. Tumor resection, typically approached endoscopically or by craniotomy, is commonly applied with adjuvant irradiation treatment. More recent studies are looking toward the use of transsphenoidal resection, particularly for the pediatric community in which craniopharyngiomas display higher incidence rates [8].

## Conclusions

Craniopharyngiomas have a high recurrence rate, however, recurrence is usually abutting the site of the original tumor bed. When ectopic recurrence does occur, it is thought to be the result of direct seeding through the cerebral spinal fluid during surgery. In this patient, the original tumor site was in the suprasellar region and tumor recurrence occurred in the right lateral ventricle, consistent with this observation in the literature. A meticulous surgical procedure, along with expectant lifelong management for patients diagnosed with craniopharyngiomas is an essential characteristic of treatment. In patients with a history of a craniopharyngioma presenting with neurological symptoms such as a headache, visual changes, or altered mentation, tumor recurrence should be considered. In the absence of a lesion in the sellar or suprasellar areas, radiologists and clinicians should together evaluate for possible ectopic recurrence.
